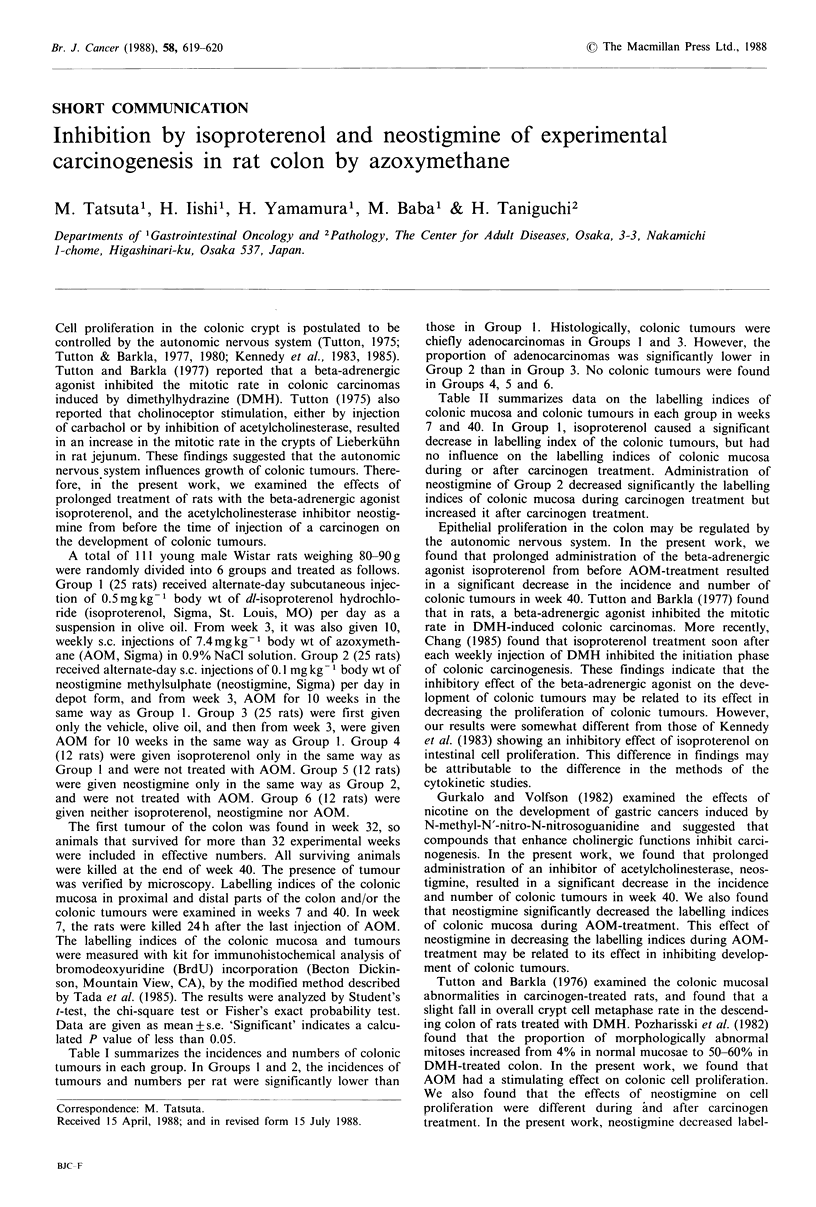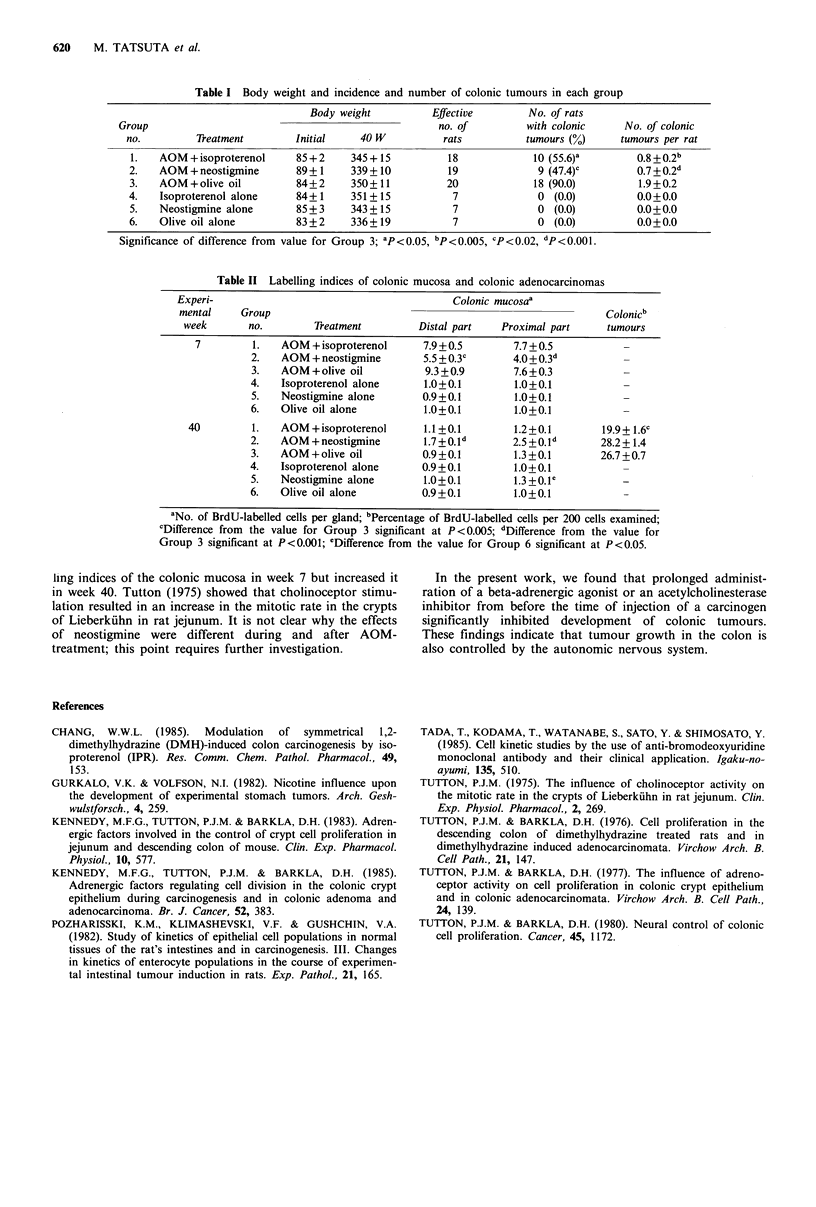# Inhibition by isoproterenol and neostigmine of experimental carcinogenesis in rat colon by azoxymethane.

**DOI:** 10.1038/bjc.1988.271

**Published:** 1988-11

**Authors:** M. Tatsuta, H. Iishi, H. Yamamura, M. Baba, H. Taniguchi

**Affiliations:** Department of Gastrointestinal Oncology, Center for Adult Disease, Osaka, Japan.


 © The Macmillan Press Ltd., 1988

**SHORT COMMUNICATION**

### Inhibition by isoproterenol and neostigmine of experimental carcinogenesis in rat colon by azoxymethane

M. Tatsuta[1], H. Iishi[1], H. Yamamura[1], M. Baba[1] & H. Taniguchi[2]

*Departments of [1]Gastrointestinal Oncology and [2]Pathology, The Center for Adult Diseases, Osaka, 3-3, Nakamichi 1-chome, Higashinari-ku, Osaka 537, Japan.*

Cell proliferation in the colonic crypt is postulated to be controlled by the autonomic nervous system (Tutton, 1975; Tutton & Barkla, 1977, 1980; Kennedy *et al.*, 1983, 1985). Tutton and Barkla (1977) reported that a beta-adrenergic agonist inhibited the mitotic rate in colonic carcinomas induced by dimethylhydrazine (DMH). Tutton (1975) also reported that cholinoceptor stimulation, either by injection of carbachol or by inhibition of acetylcholinesterase, resulted in an increase in the mitotic rate in the crypts of Lieberkühn in rat jejunum. These findings suggested that the autonomic nervous system influences growth of colonic tumours. Therefore, in the present work, we examined the effects of prolonged treatment of rats with the beta-adrenergic agonist isoproterenol, and the acetylcholinesterase inhibitor neostigmine from before the time of injection of a carcinogen on the development of colonic tumours.

A total of 111 young male Wistar rats weighing 80–90 g were randomly divided into 6 groups and treated as follows. Group 1 (25 rats) received alternate-day subcutaneous injection of 0.5 mg kg$^{-1}$ body wt of *dl*-isoproterenol hydrochloride (isoproterenol, Sigma, St. Louis, MO) per day as a suspension in olive oil. From week 3, it was also given 10, weekly s.c. injections of 7.4 mg kg$^{-1}$ body wt of azoxymethane (AOM, Sigma) in 0.9% NaCl solution. Group 2 (25 rats) received alternate-day s.c. injections of 0.1 mg kg$^{-1}$ body wt of neostigmine methylsulphate (neostigmine, Sigma) per day in depot form, and from week 3, AOM for 10 weeks in the same way as Group 1. Group 3 (25 rats) were first given only the vehicle, olive oil, and then from week 3, were given AOM for 10 weeks in the same way as Group 1. Group 4 (12 rats) were given isoproterenol only in the same way as Group 1 and were not treated with AOM. Group 5 (12 rats) were given neostigmine only in the same way as Group 2, and were not treated with AOM. Group 6 (12 rats) were given neither isoproterenol, neostigmine nor AOM.

The first tumour of the colon was found in week 32, so animals that survived for more than 32 experimental weeks were included in effective numbers. All surviving animals were killed at the end of week 40. The presence of tumour was verified by microscopy. Labelling indices of the colonic mucosa in proximal and distal parts of the colon and/or the colonic tumours were examined in weeks 7 and 40. In week 7, the rats were killed 24 h after the last injection of AOM. The labelling indices of the colonic mucosa and tumours were measured with kit for immunohistochemical analysis of bromodeoxyuridine (BrdU) incorporation (Becton Dickinson, Mountain View, CA), by the modified method described by Tada *et al.* (1985). The results were analyzed by Student's *t*-test, the chi-square test or Fisher's exact probability test. Data are given as mean ± s.e. 'Significant' indicates a calculated $P$ value of less than 0.05.

Table I summarizes the incidences and numbers of colonic tumours in each group. In Groups 1 and 2, the incidences of tumours and numbers per rat were significantly lower than those in Group 1. Histologically, colonic tumours were chiefly adenocarcinomas in Groups 1 and 3. However, the proportion of adenocarcinomas was significantly lower in Group 2 than in Group 3. No colonic tumours were found in Groups 4, 5 and 6.

Table II summarizes data on the labelling indices of colonic mucosa and colonic tumours in each group in weeks 7 and 40. In Group 1, isoproterenol caused a significant decrease in labelling index of the colonic tumours, but had no influence on the labelling indices of colonic mucosa during or after carcinogen treatment. Administration of neostigmine of Group 2 decreased significantly the labelling indices of colonic mucosa during carcinogen treatment but increased it after carcinogen treatment.

Epithelial proliferation in the colon may be regulated by the autonomic nervous system. In the present work, we found that prolonged administration of the beta-adrenergic agonist isoproterenol from before AOM-treatment resulted in a significant decrease in the incidence and number of colonic tumours in week 40. Tutton and Barkla (1977) found that in rats, a beta-adrenergic agonist inhibited the mitotic rate in DMH-induced colonic carcinomas. More recently, Chang (1985) found that isoproterenol treatment soon after each weekly injection of DMH inhibited the initiation phase of colonic carcinogenesis. These findings indicate that the inhibitory effect of the beta-adrenergic agonist on the development of colonic tumours may be related to its effect in decreasing the proliferation of colonic tumours. However, our results were somewhat different from those of Kennedy *et al.* (1983) showing an inhibitory effect of isoproterenol on intestinal cell proliferation. This difference in findings may be attributable to the difference in the methods of the cytokinetic studies.

Gurkalo and Volfson (1982) examined the effects of nicotine on the development of gastric cancers induced by N-methyl-N′-nitro-N-nitrosoguanidine and suggested that compounds that enhance cholinergic functions inhibit carcinogenesis. In the present work, we found that prolonged administration of an inhibitor of acetylcholinesterase, neostigmine, resulted in a significant decrease in the incidence and number of colonic tumours in week 40. We also found that neostigmine significantly decreased the labelling indices of colonic mucosa during AOM-treatment. This effect of neostigmine in decreasing the labelling indices during AOM-treatment may be related to its effect in inhibiting development of colonic tumours.

Tutton and Barkla (1976) examined the colonic mucosal abnormalities in carcinogen-treated rats, and found that a slight fall in overall crypt cell metaphase rate in the descending colon of rats treated with DMH. Pozharisski *et al.* (1982) found that the proportion of morphologically abnormal mitoses increased from 4% in normal mucosae to 50–60% in DMH-treated colon. In the present work, we found that AOM had a stimulating effect on colonic cell proliferation. We also found that the effects of neostigmine on cell proliferation were different during and after carcinogen treatment. In the present work, neostigmine decreased label-

Correspondence: M. Tatsuta.
Received 15 April, 1988; and in revised form 15 July 1988.

**Table I** Body weight and incidence and number of colonic tumours in each group

| Group no. | Treatment | Body weight: Initial | Body weight: 40 W | Effective no. of rats | No. of rats with colonic tumours (%) | No. of colonic tumours per rat |
|---|---|---|---|---|---|---|
| 1. | AOM + isoproterenol | 85+2 | 345+15 | 18 | 10 (55.6)[a] | 0.8±0.2[b] |
| 2. | AOM + neostigmine | 89±1 | 339±10 | 19 | 9 (47.4)[c] | 0.7±0.2[d] |
| 3. | AOM + olive oil | 84±2 | 350±11 | 20 | 18 (90.0) | 1.9±0.2 |
| 4. | Isoproterenol alone | 84±1 | 351±15 | 7 | 0 (0.0) | 0.0±0.0 |
| 5. | Neostigmine alone | 85±3 | 343±15 | 7 | 0 (0.0) | 0.0±0.0 |
| 6. | Olive oil alone | 83±2 | 336±19 | 7 | 0 (0.0) | 0.0±0.0 |

Significance of difference from value for Group 3; [a]$P<0.05$, [b]$P<0.005$, [c]$P<0.02$, [d]$P<0.001$.

**Table II** Labelling indices of colonic mucosa and colonic adenocarcinomas

| Experimental week | Group no. | Treatment | Colonic mucosa[a]: Distal part | Colonic mucosa[a]: Proximal part | Colonic[b] tumours |
|---|---|---|---|---|---|
| 7 | 1. | AOM + isoproterenol | 7.9±0.5 | 7.7±0.5 | – |
| | 2. | AOM + neostigmine | 5.5±0.3[c] | 4.0±0.3[d] | – |
| | 3. | AOM + olive oil | 9.3±0.9 | 7.6±0.3 | – |
| | 4. | Isoproterenol alone | 1.0±0.1 | 1.0±0.1 | – |
| | 5. | Neostigmine alone | 0.9±0.1 | 1.0±0.1 | – |
| | 6. | Olive oil alone | 1.0±0.1 | 1.0±0.1 | – |
| 40 | 1. | AOM + isoproterenol | 1.1±0.1 | 1.2±0.1 | 19.9±1.6[c] |
| | 2. | AOM + neostigmine | 1.7±0.1[d] | 2.5±0.1[d] | 28.2±1.4 |
| | 3. | AOM + olive oil | 0.9±0.1 | 1.3±0.1 | 26.7±0.7 |
| | 4. | Isoproterenol alone | 0.9±0.1 | 1.0±0.1 | – |
| | 5. | Neostigmine alone | 1.0±0.1 | 1.3±0.1[e] | – |
| | 6. | Olive oil alone | 0.9±0.1 | 1.0±0.1 | – |

[a]No. of BrdU-labelled cells per gland; [b]Percentage of BrdU-labelled cells per 200 cells examined; [c]Difference from the value for Group 3 significant at $P<0.005$; [d]Difference from the value for Group 3 significant at $P<0.001$; [e]Difference from the value for Group 6 significant at $P<0.05$.

ling indices of the colonic mucosa in week 7 but increased it in week 40. Tutton (1975) showed that cholinoceptor stimulation resulted in an increase in the mitotic rate in the crypts of Lieberkühn in rat jejunum. It is not clear why the effects of neostigmine were different during and after AOM-treatment; this point requires further investigation.

In the present work, we found that prolonged administration of a beta-adrenergic agonist or an acetylcholinesterase inhibitor from before the time of injection of a carcinogen significantly inhibited development of colonic tumours. These findings indicate that tumour growth in the colon is also controlled by the autonomic nervous system.

### References

CHANG, W.W.L. (1985). Modulation of symmetrical 1,2-dimethylhydrazine (DMH)-induced colon carcinogenesis by isoproterenol (IPR). *Res. Comm. Chem. Pathol. Pharmacol.*, **49**, 153.

GURKALO, V.K. & VOLFSON, N.I. (1982). Nicotine influence upon the development of experimental stomach tumors. *Arch. Geshwulstforsch.*, **4**, 259.

KENNEDY, M.F.G., TUTTON, P.J.M. & BARKLA, D.H. (1983). Adrenergic factors involved in the control of crypt cell proliferation in jejunum and descending colon of mouse. *Clin. Exp. Pharmacol. Physiol.*, **10**, 577.

KENNEDY, M.F.G., TUTTON, P.J.M. & BARKLA, D.H. (1985). Adrenergic factors regulating cell division in the colonic crypt epithelium during carcinogenesis and in colonic adenoma and adenocarcinoma. *Br. J. Cancer*, **52**, 383.

POZHARISSKI, K.M., KLIMASHEVSKI, V.F. & GUSHCHIN, V.A. (1982). Study of kinetics of epithelial cell populations in normal tissues of the rat's intestines and in carcinogenesis. III. Changes in kinetics of enterocyte populations in the course of experimental intestinal tumour induction in rats. *Exp. Pathol.*, **21**, 165.

TADA, T., KODAMA, T., WATANABE, S., SATO, Y. & SHIMOSATO, Y. (1985). Cell kinetic studies by the use of anti-bromodeoxyuridine monoclonal antibody and their clinical application. *Igaku-no-ayumi*, **135**, 510.

TUTTON, P.J.M. (1975). The influence of cholinoceptor activity on the mitotic rate in the crypts of Lieberkühn in rat jejunum. *Clin. Exp. Physiol. Pharmacol.*, **2**, 269.

TUTTON, P.J.M. & BARKLA, D.H. (1976). Cell proliferation in the descending colon of dimethylhydrazine treated rats and in dimethylhydrazine induced adenocarcinomata. *Virchow Arch. B. Cell Path.*, **21**, 147.

TUTTON, P.J.M. & BARKLA, D.H. (1977). The influence of adrenoceptor activity on cell proliferation in colonic crypt epithelium and in colonic adenocarcinomata. *Virchow Arch. B. Cell Path.*, **24**, 139.

TUTTON, P.J.M. & BARKLA, D.H. (1980). Neural control of colonic cell proliferation. *Cancer*, **45**, 1172.